# Tissue specific innate immune responses impact viral infection in Drosophila

**DOI:** 10.1371/journal.ppat.1012672

**Published:** 2024-11-04

**Authors:** Elisha Segrist, Steven Miller, Beth Gold, Yue Li, Sara Cherry

**Affiliations:** 1 National Institute of General Medical Sciences, National Institutes of Health, Bethesda, Maryland, United States of America; 2 Department of Pathology and Laboratory Medicine, Perelman School of Medicine, University of Pennsylvania, Philadelphia, Pennsylvania, United States of America; University of Massachusetts, Worcester, UNITED STATES OF AMERICA

## Abstract

All organisms sense and respond to pathogenic challenge. Tissue-specific responses are required to combat pathogens infecting distinct cell types. Cyclic dinucleotides (CDNs) are produced endogenously downstream of pathogen recognition or by pathogens themselves which bind to STING to activate NF-kB-dependent antimicrobial gene expression programs. It remains unknown whether there are distinct immune responses to CDNs in *Drosophila* tissues. Here, we investigated tissue specific CDN-STING responses and uncovered differences in gene-induction patterns across tissues that play important roles in viral infections. Using tissue-and cell-specific genetic studies we found that *dSTING* in the fat body controls CDN-induced expression of dSTING-regulated gene 1 (*Srg1*) but not dSTING-regulated gene 2 (*Srg2*) or 3 (*Srg3*). In contrast, the gastrointestinal tract largely controls expression of *Srg2* and *Srg3*. We found that *Srg3* is antiviral against the natural fly pathogen Drosophila C virus and the human arthropod-borne Rift Valley Fever virus (RVFV), but not other arthropod-borne viruses including Sindbis virus and dengue virus. Furthermore, we found that *Srg3* has an important role in controlling RVFV infection of the ovary which has important implications in understanding vertical transmission of viruses and RVFV in mosquitoes. Overall, our study underscores the importance of tissue-specific responses in antiviral immunity and highlights the complex tissue regulation of the CDN-STING pathway.

## Author summary

The cyclic dinucleotide (CDN)-STING pathway (CDN-STING pathway) is a highly conserved innate immune pathway that stimulates the production of antiviral genes in response to infection. We found that diverse fly tissues respond to CDN stimulation, but dSTING regulates distinct subsets of genes across tissues. We also found that the CDN regulated gene, dSTING regulated gene 3 (*Srg3*), was antiviral against the human arbovirus Rift Valley Fever virus but not dengue or Sindbis virus. Furthermore, we found that *Srg3* has an important role in controlling RVFV infection of the ovary which has important implications in vertical transmission of viruses such as RVFV. These findings highlight the tissue-specific activity of the CDN-STING pathway and emphasize the importance of understanding how different tissues sense and integrate immune signals during pathogenic challenge.

## Introduction

Viral infections present a serious challenge to all animals, including *Drosophila*, which exclusively use innate immune pathways to recognize and combat pathogens. Viruses infect organisms through distinct routes and have diverse tissue tropisms. The highly conserved STING pathway is activated by binding cyclic dinucleotides (CDNs) [1–4]; reviewed in [5]. CDNs can be produced exogenously by bacteria or endogenously by host enzymes including cGAS-like receptors (cGLRs) [6–9]; reviewed in [10]. Once activated, STING can induce NF-kB signaling leading to robust gene expression changes [8,11,12]; reviewed in [13].

*Drosophila* STING (dSTING) is antiviral against diverse viruses during systemic infection including Drosophila C virus (DCV), cricket paralysis virus (CrPV), flock house virus (FHV), vesicular stomatitis virus (VSV) and Kallithea virus (KV) [7], [14–20]; reviewed in [21]. Only a few studies have explored dSTING’s antiviral activity in specific tissues. One found that dSTING protects the brain from systemic Zika virus infection [16]. Furthermore, during oral infection, dSTING is protective against DCV and Sindbis virus (SINV) infection in intestinal epithelial cells in the intestinal tract [17]. CDNs can be produced by commensal and pathogenic bacteria [1,22] and we found that orally acquired CDNs, likely from the gut microbiota, protect the intestine from infection and induce gene expression changes in a dSTING- and NF-kB-dependent manner [17]. Furthermore, administration of CDNs systemically can protect animals from virus infection including DCV [14,17]. *Drosophila* cGAS orthologs (cGLR1 and cGLR2) are an endogenous source of CDNs and these genes can impact systemic viral infection [6–9].

While most studies have explored infection in whole flies, studies of dSTING activity in the brain and intestine suggest tissue-specific regulation of dSTING-dependent antiviral responses [16,17]. For example, NF-kB gene expression controls antiviral activity in the gut while dSTING-dependent autophagy controls infection in the brain [16,17]. There are additional data that suggest CDN-dSTING signaling can have tissue-specific antiviral activity. For example, dSTING is antiviral during systemic infection of DCV, but not the arthropod-borne virus SINV [14,16,17]. In contrast, dSTING provides antiviral protection against both DCV and SINV during oral infection [17]. These results highlight the need to understand CDN-STING signaling in antiviral protection across tissues.

Treatment with CDNs induces the canonical dSTING transcriptional targets *Srg1*, *Srg2* and *Srg3* in an NF-kB (*Relish*) dependent manner [6,14,15]. By assessing CDN-dSTING activity in distinct tissues we found that these canonical targets are differentially expressed at baseline and upon stimulation. Furthermore, using a genetic approach we selectively depleted *dSTING* in tissues known to be targets of viral infection and explored the requirements for CDN-dependent gene induction [16,17,23–25]. This reveals tissue-specific regulation of dSTING targets where dSTING signaling in the gut largely controls CDN-induced expression of *Srg2* while dSTING signaling in the fat body controls *Srg1*. *Srg3* has complex regulation where it is induced by many tissues, but the gut is a major producer. Furthermore, we find that specific loss of dSTING in the fat body leads to both increased DCV infection and decreased survival. Lastly, we find that *Srg3* but not *Srg1* or *Srg2* is antiviral against systemic DCV challenge as loss of *Srg3* leads to both an increase in DCV replication and a decrease in survival upon DCV infection. Infection studies with disparate viruses revealed *Srg3’s* antiviral activity is selective as it restricts the human arthropod-borne Rift Valley Fever virus (RVFV) but not SINV or dengue virus (DENV). Furthermore, we found that *Srg3* has an important role in controlling RVFV infection of the ovary which has important implications in vertical transmission of viruses and potentially RVFV in mosquitoes [26,27]; reviewed in [28]. This work highlights the importance of an organ and cell-type specific approach in investigating antiviral immunity and further delineates the mechanisms of CDN-STING signaling in *Drosophila*.

## Results

### dSTING displays tissue specific antiviral activity during systemic DCV infection

**Fig 1:**
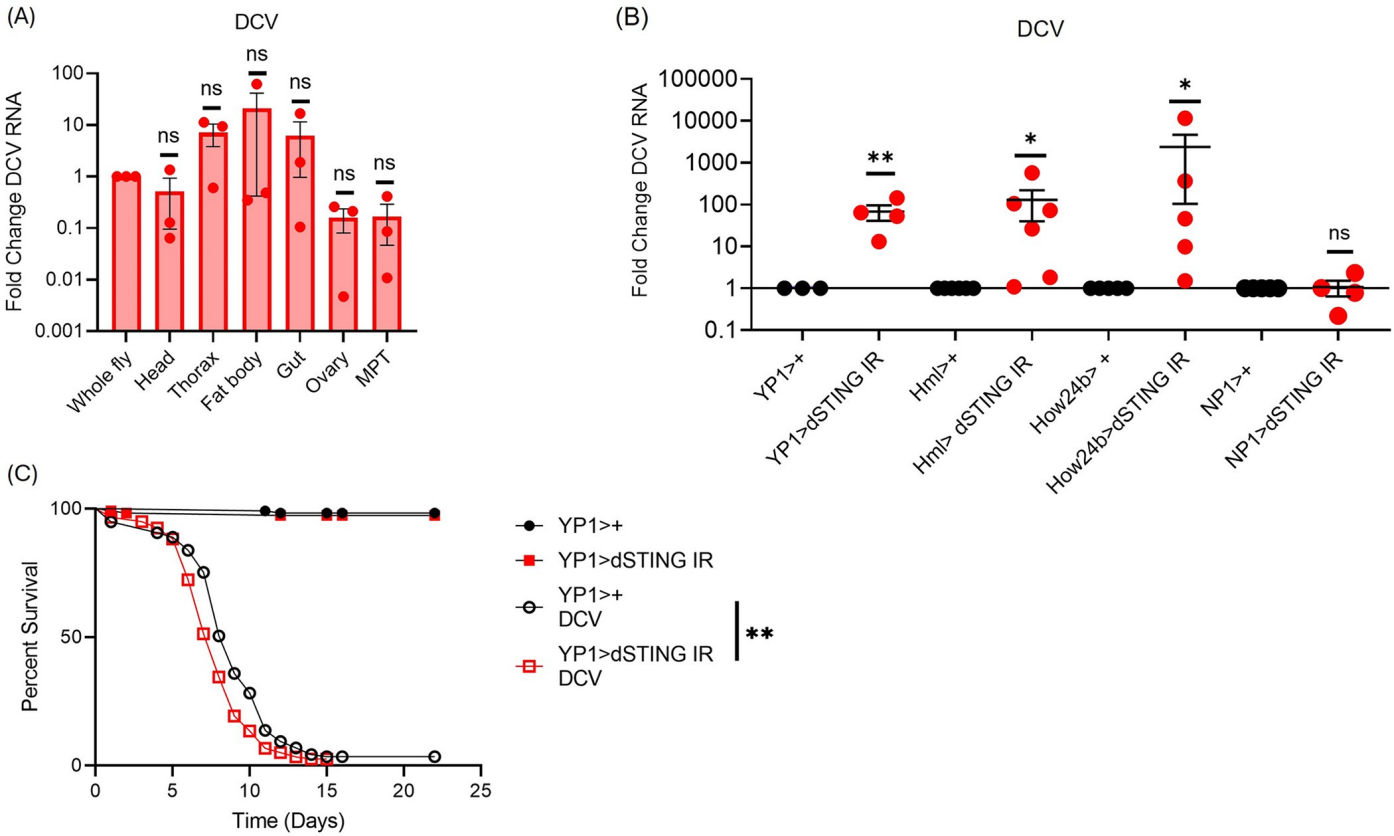
*dSTING* in the fat body, visceral muscle and hemocytes controls systemic DCV infection. (A) Wild type (w1118) flies were systemically infected with DCV and groups of 5 whole flies and 15 individual tissues were collected at 24hpi. (B) Flies of the indicated genotype were systemically infected with DCV and groups of 5 whole flies were collected 4 days post infection. DCV levels were quantified by RT-qPCR and normalized to controls relative to the housekeeping gene *rp49*. n = 3–6. Each dot represents an independent experiment (A,B) of 5 pooled whole animals or (A) 15 pooled individual tissues with mean ± SEM shown. A one sample t-test was performed to determine statistical significance. A Grubbs’ test was performed to remove statistical outliers. (C) Survival curves for uninfected or DCV infected control (*YP1>+*) and *dSTING* fat body depleted (*YP1>dSTING IR*) flies. Eight independent experiments were performed for a total of n = 233 uninfected and n = 224 infected flies across all eight replicates. Significance was determined by a logrank test. ns, not significant * p<0.05, ** p<0.01.

DCV infects diverse tissues during systemic infection, including the fat body and muscle [24]. Indeed, at 24h post infection we detected DCV infection in head, thorax, fat body, gut, ovary, and Malpighian tubules (MPT) (Fig 1A). Since dSTING controls systemic and oral DCV infection, we systematically tested which tissues are sufficient to control DCV infection via dSTING [14,17]. Therefore, we depleted dSTING in the fat body (*YP1>dSTING IR*), hemocytes (*Hml>dSTING IR*), visceral muscle (*How24b>dSTING IR*), and enterocytes (*NP1>dSTING IR*) and challenged flies with DCV. We found that loss of dSTING in the fat body, visceral muscle and hemocytes resulted in a significant increase in systemic DCV RNA levels. However, loss of dSTING in enterocytes had no influence on systemic DCV infection (Fig 1B). Additionally, we performed survival studies with flies depleted of dSTING either in the fat body or thorax. We found modestly decreased time to death in animals with dSTING depleted in the fat body (*YP1>dSTING IR*) as compared to control flies (*YP1>+*) upon DCV challenge (Fig 1C) but no change in survival for flies depleted in the thorax (*How24b>dSTING IR* compared to *How24b>+*) (S1A Fig). These data suggest that dSTING’s role in protection from infection spans multiple organs, but that dSTING activity at certain body sites, like the fat body, play a significant role in protecting the whole organism from lethality caused by DCV infection.

### CDN induced gene expression changes are dSTING- and NF-kB-dependent

**Fig 2:**
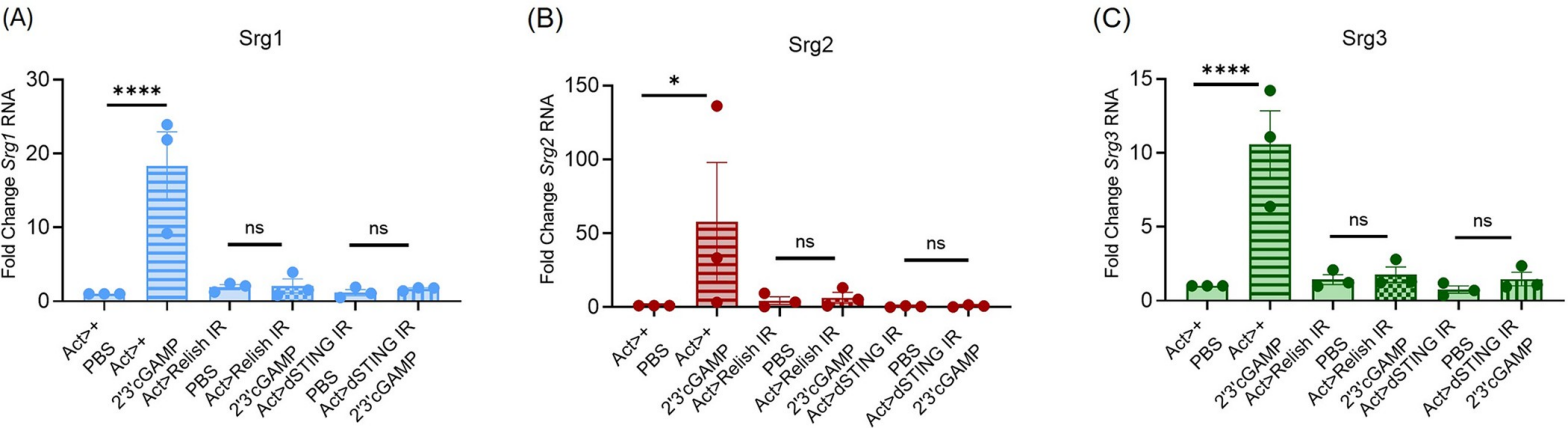
CDN induced gene expression changes are dSTING- and NF-kB dependent. (A-C) Control (*Act>+*), NF-kB (*Relish*) depleted (*Act>Relish IR*), and *dSTING* depleted (*Act>dSTING IR*) flies were treated with PBS or 2’3’cGAMP and groups of 5 whole flies were collected 6 hours later. Indicated genes (*Srg1-3*) were quantified by RT-qPCR and normalized to controls relative to the housekeeping gene *rp49*. n = 3. Each dot represents an independent experiment of 5 pooled whole animals with mean ± SEM shown. A one-way Anova with multiple comparisons was performed to determine statistical significance. ns, not significant, * p<0.05, **** p<0.0001.

Genes regulated by CDN-dSTING signaling have been characterized and include the canonical targets *Srg1*, *Srg2* and *Srg3* [6,14,15]. Studies have also shown that induction of these targets is dependent on the NF-kB transcription factor Relish during CDN treatment as well as DCV, CrPV [14] and VSV [15] infection. We first confirmed that induction of these canonical targets was dSTING- and NF-kB-dependent downstream of systemic CDN treatment. We used an Actin driver to ubiquitously deplete dSTING (*Act>dSTING IR*) or NF-kB (*Relish*) (*Act> Relish IR*) and systemically treated animals with 2’3’cGAMP. We monitored gene expression changes of the canonical dSTING activated genes *Srg1*, *Srg2* and *Srg3* 6 hours later by RT-qPCR. In agreement with previous findings, we found dSTING and NF-kB (*Relish*) are necessary for 2’3’cGAMP-mediated induction of *Srg1*, *Srg2* and *Srg3* expression in whole animals (Fig 2A–2C) [15,17].

dSTING-dependent autophagy controls ZIKV in the brain of flies, and autophagy can control gene expression [16,29]. Therefore, we tested if CDN induced gene expression changes are dependent on autophagy using the autophagy gene *ATG16*. We found treatment with 2’3’cGAMP induced expression of *Srg2* in control (*Act>+*) or *Atg16* depleted (*Act>Atg16 IR*) flies (S1B Fig).

The RNAi pathway is known to have antiviral activity and can also regulate gene expression [30,31]; reviewed in [32]. Thus, we tested if the RNAi pathway impacts expression of dSTING gene targets. We monitored *Srg2* RNA levels in control (w1118) or *Ago2* mutant flies (*Ago2 -/-*) 6 hours after CDN treatment. We found that 2’3’cGAMP induced expression of *Srg2* in control and *Ago2* mutant flies (S1C Fig). Altogether, these data suggest CDN-dependent gene induction during systemic exposure occurs independently of autophagy or RNAi pathways.

### Srg1, Srg2 and Srg3 gene expression varies across tissues

**Fig 3:**
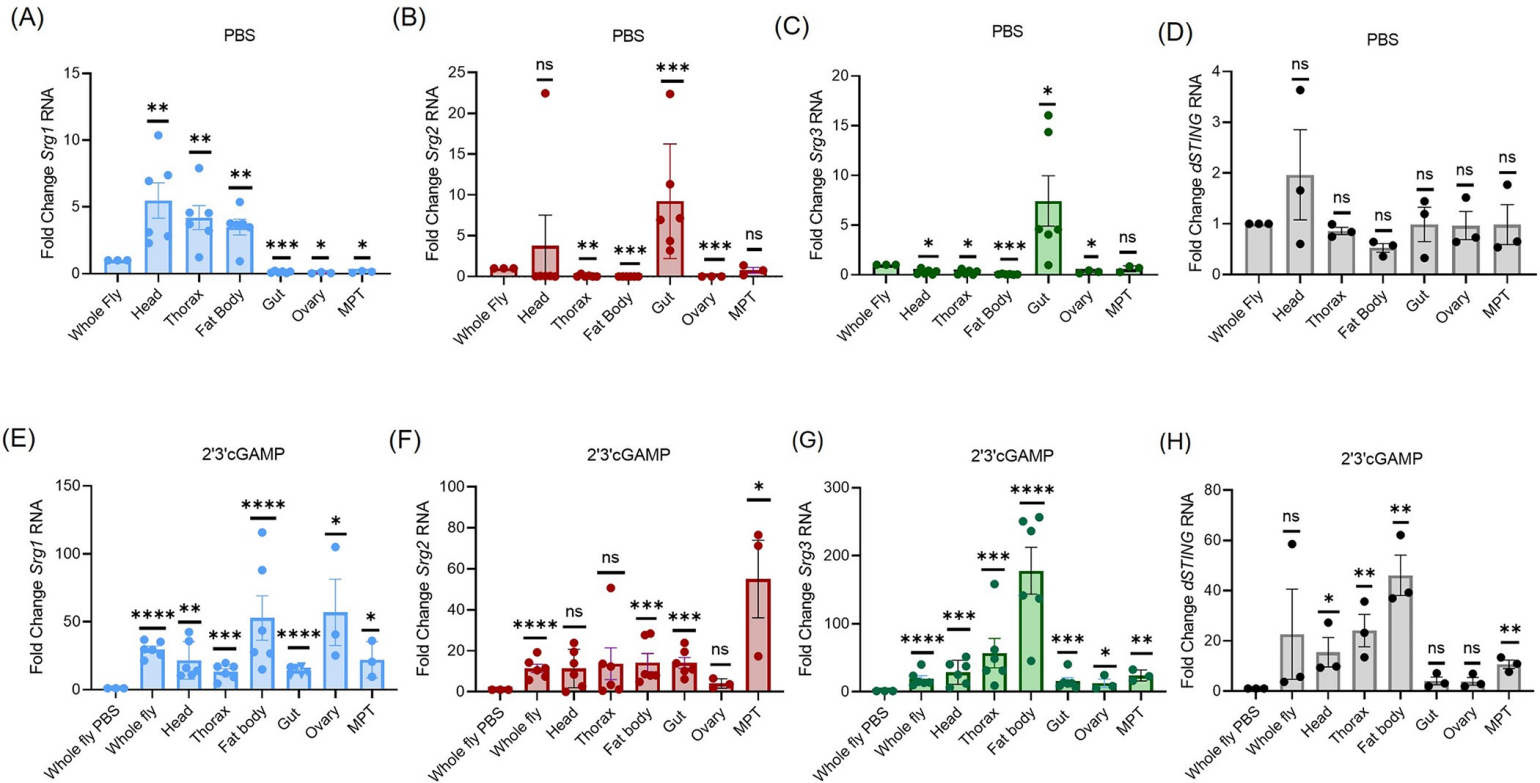
*Srg1-3* expression is differentially regulated across tissues. (A-H) Wild type (w1118) flies were systemically treated with either PBS or 2’3’cGAMP and 5 whole flies were collected or 15 flies were dissected for specific tissues (head, thorax, fat body, gut, ovary, Malpighian tubules (MPT)) 6 hours later. Indicated genes *(Srg1-3*, *dSTING*) were quantified by RT-qPCR. (A-D) RNA levels of *Srg1-3* and *dSTING* were quantified in PBS treated animals and expression was normalized to whole flies relative to *rp49*. n = 3–6. (E-H) Tissue specific expression of *Srg1-3* and *dSTING* after 2’3’cGAMP treatment were normalized to PBS treated tissues relative to *rp49*. n = 3–6. (A-H) Each dot represents an independent experiment of 5 pooled whole flies or 15 pooled tissues with mean ± SEM shown. A one sample t-test was performed to determine statistical significance. ns, not significant, * p<0.05, ** p<0.01, *** p>0.001, **** p<0.0001.

Next, we set out to determine how expression of these dSTING regulated genes is controlled in diverse tissues. To determine basal expression of *Srg1-3* and *dSTING* across tissues, we quantified their RNA levels in whole animals and in individual tissues (gut, fat body, thorax, head, ovary and Malpighian tubules) by RT-qPCR 6 hours after vehicle (PBS) treatment. Relative to whole flies, the intestine had lower baseline expression of *Srg1* but had higher expression of *Srg2* and *Srg3* (Fig 3A–3C). In contrast, the thorax and fat body had higher baseline expression of *Srg1* but had lower expression of *Srg2* and *Srg3* relative to whole flies. While baseline expression of these dSTING-regulated genes varied across tissues, all tissues induced transcription of *Srg1-3* in response to systemic treatment with 2’3’cGAMP, albeit to different levels (Fig 3E–3G). *dSTING* expression did not vary across tissues as compared to whole flies, even after CDN stimulation (Fig 3D and 3H). *Drosophila* naturally produce many different antiviral CDN species [8], therefore we evaluated whether a different endogenous CDN, 2’3’cdiAMP [9], would elicit transcriptional responses similar to 2’3’cGAMP across tissues. As with 2’3’cGAMP, we found that all tissues induced transcription of at least one of the dSTING regulated genes in response to stimulation with 2’3’cdiAMP (S2A–S2D Fig).

We also tested if virus challenge could activate expression of dSTING and its regulated genes. To do this, we systemically infected wild type flies with PBS or DCV and measured *Srg1-3* and *dSTING* RNA levels across tissues by RT-qPCR at 6 hours and 24 hours post infection. We found that systemic infection of DCV did not significantly induce *Srg1-3* or *dSTING* at either timepoint in any tissue relative to PBS challenged animals (S3A–S3H Fig).

### STING in the visceral muscle does not control systemic expression of CDN induced genes

**Fig 4:**
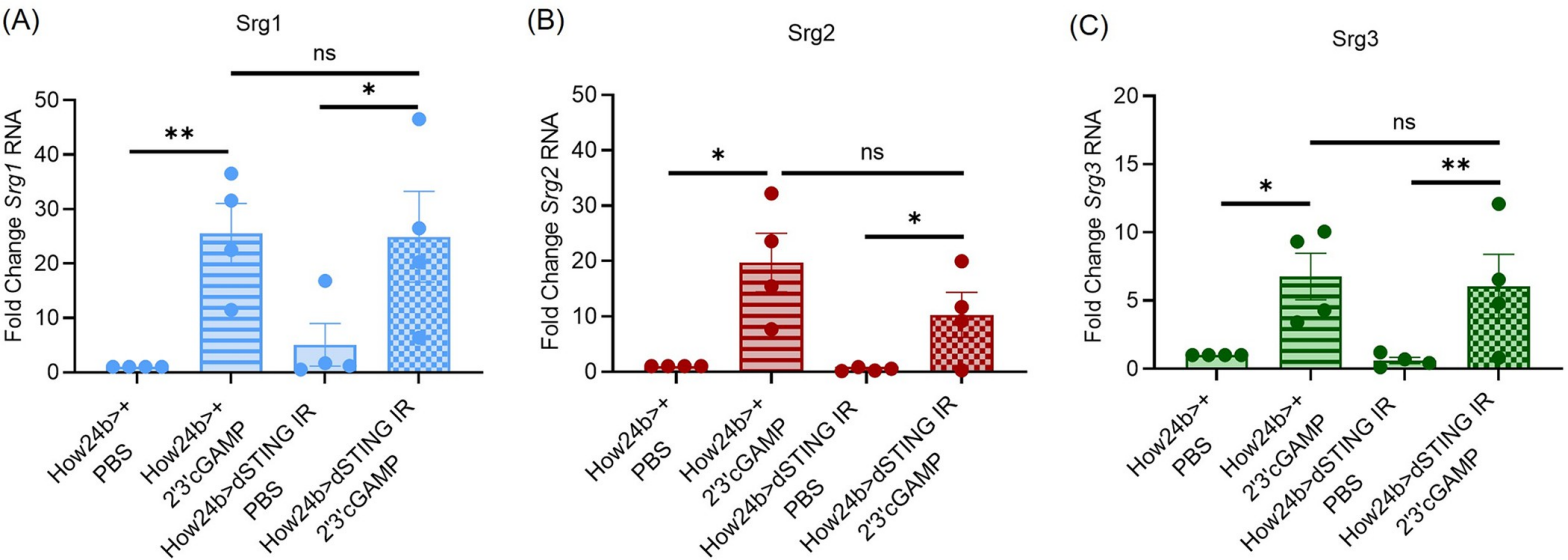
*dSTING* expression in muscle does not control gene expression changes induced by 2’3’cGAMP in whole animals. (A-C) Control (*How24b>+*) or *dSTING* visceral muscle depleted (*How24b>dSTING IR*) flies were treated with PBS or 2’3’cGAMP and groups of 5 whole flies were collected 6 hours later. Indicated genes (*Srg1-3*) were quantified by RT-qPCR and normalized to controls relative to the housekeeping gene *rp49*. n = 4. Each dot represents an independent experiment of 5 pooled whole animals with mean ± SEM shown. A one-way Anova with multiple comparisons was performed to determine statistical significance. ns, not significant, * p<0.05, ** p<0.01.

As muscle is a major site of DCV replication [24], and a site where dSTING controls infection (Fig 1B), we investigated how dSTING senses and responds to CDNs at this site. We treated control (*How24b>+*) and dSTING visceral muscle depleted (*How24b>dSTING IR*) animals with 2’3’cGAMP and quantified expression of *Srg1-3* RNA in whole animals 6 hours later. We found that loss of dSTING in the muscle did not alter CDN-dependent increases in *Srg1-3* RNA expression (Fig 4A–4C). This indicates that visceral muscle dSTING does not contribute to CDN-induced expression of *Srg1-3* found in whole animals.

### dSTING in the fat body controls systemic expression of Srg1, but not Srg2 or Srg3

**Fig 5:**
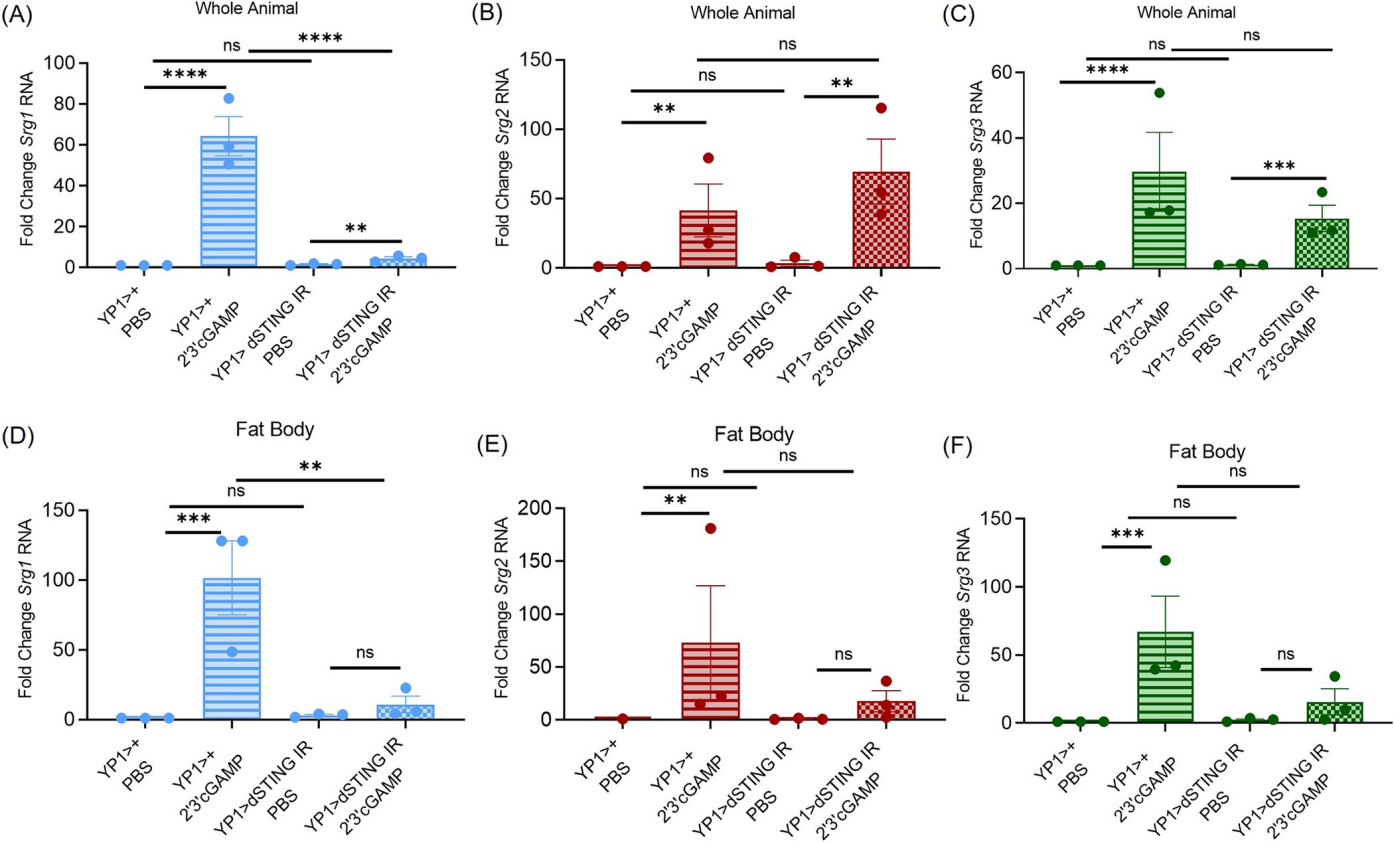
Fat body *dSTING* controls systemic expression of *Srg1*, but not *Srg2* or *Srg3*. (A-F) Control (*YP1>+*) and *dSTING* fat body depleted (*YP1>dSTING IR*) flies were treated with PBS or 2’3’cGAMP and either (A-C) 5 whole flies or (D-F) 15 fat bodies were collected 6 hours later. Indicated genes (*Srg1-3*) were quantified by RT-qPCR and normalized to controls relative to the housekeeping gene *rp49*. n = 3. Each dot represents an independent experiment (A-C) of 5 pooled whole animals and (D-F) of 15 pooled fat bodies with mean ± SEM shown. A one-way Anova with multiple comparisons was performed to determine statistical significance. ns, not significant, ** p<0.01, *** p<0.001, **** p<0.0001.

The fat body is also a major site of DCV replication [24] and we found that dSTING expression in the fat body is important for protection from DCV infection (Fig 1B and 1C). Therefore, we also investigated whether dSTING protects the fat body from infection via regulation of antiviral gene expression programs. We injected control (*YP1>+*) and dSTING fat body depleted (*YP1>dSTING IR*) flies with PBS or 2’3’cGAMP and measured expression of *Srg1-3*, by RT-qPCR 6 hours later in whole animals. We found that loss of dSTING specifically in the fat body reduced systemic *Srg1* levels (Fig 5A) but not *Srg2* or *Srg3* (Fig 5B and 5C). This led us to monitor the expression of these genes in the abdominal fat body directly. We found that dSTING in the fat body is required for 2’3’cGAMP induced expression of all three genes, *Srg1-3* (Fig 5D–5F). This suggests that dSTING controls expression of *Srg2* and *Srg3* within the fat body, but that other tissues are a major source of systemic *Srg2* and *Srg3* RNA in response to CDN stimulation.

### dSTING in enterocytes is required for systemic Srg2 and Srg3 induction

**Fig 6:**
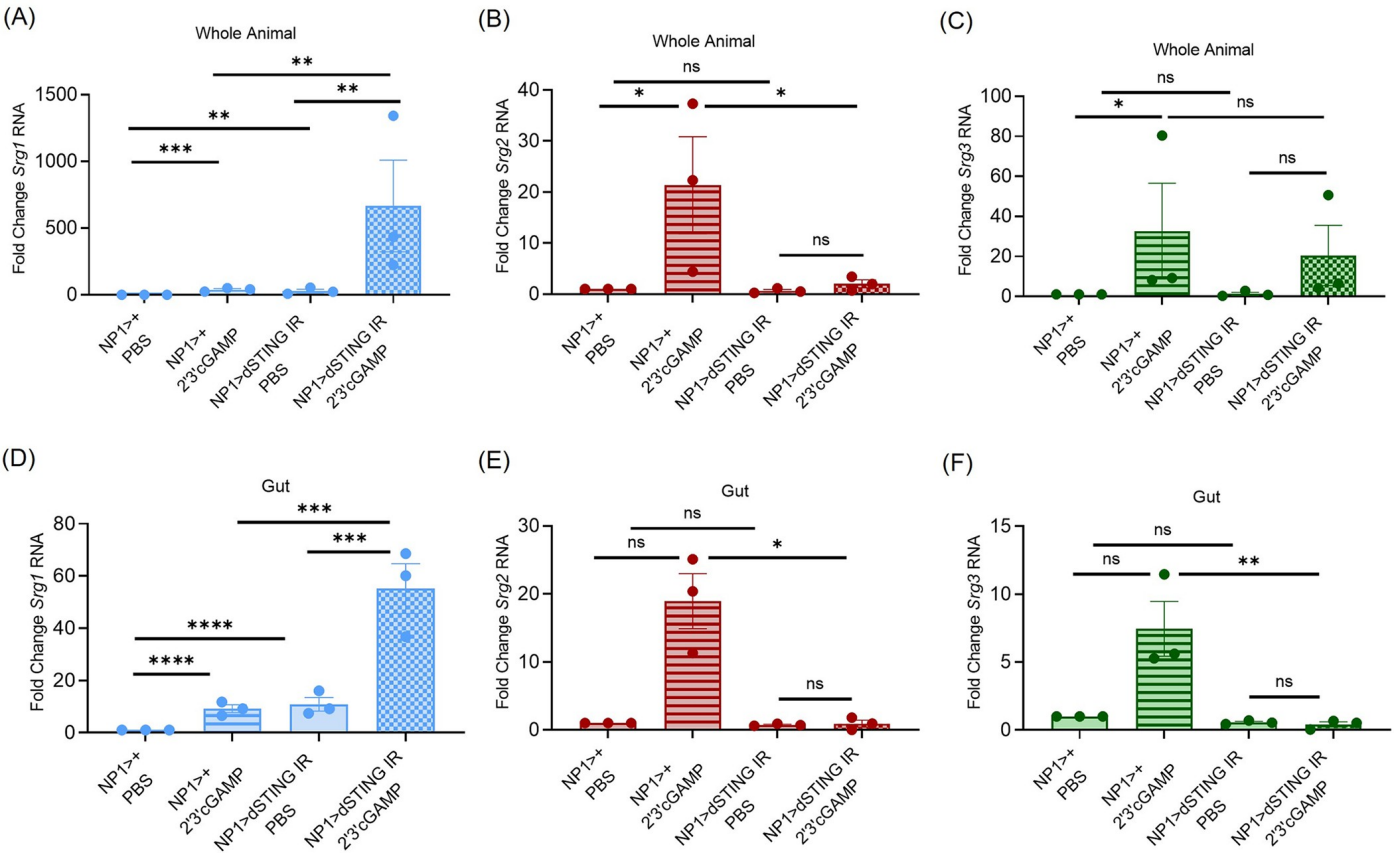
*dSTING* expression in enterocytes controls systemic expression of *Srg2* and negatively regulates *Srg1*. (A-F) Control (*NP1>+*) and *dSTING* gut depleted (*NP1>dSTING IR*) flies were treated with PBS or 2’3’cGAMP and (A-C) 5 whole flies or (D-F) 15 guts were collected 6 hours later. Indicated genes *(Srg1-3*) were quantified by RT-qPCR and normalized to controls relative to the housekeeping gene *rp49*. n = 3. Each dot represents an independent experiment of (A-C) 5 pooled whole animals and (D-F) of 15 pooled intestines with mean ± SEM shown. A one-way Anova with multiple comparisons was performed to determine statistical significance. ns, not significant, * p<0.05, ** p<0.01, *** p<0.001, **** p<0.0001.

We next determined the requirement for dSTING in the control of *Srg1-3* in the gut as dSTING is antiviral in enterocytes during oral challenge [17]. We treated control (*NP1>+*) and dSTING enterocyte depleted (*NP1>dSTING IR*) flies with PBS or 2’3’cGAMP and measured gene expression of *Srg1-3* in whole animals 6 hours post injection. Surprisingly, we found that enterocyte dSTING is a negative regulator of *Srg1* as we observed increased levels of *Srg1* upon both PBS and CDN treatment in dSTING enterocyte depleted whole flies (Fig 6A). In contrast, 2’3’cGAMP dependent expression of *Srg2* in whole animals is controlled by the gut; in the enterocyte depleted flies there is no systemic induction of *Srg2* (Fig 6B). Systemic induction of *Srg3* upon CDN administration is only partially dependent on dSTING expression in enterocytes; there is not a significant decrease systemically although the trend suggests the gut is a major producer (Fig 6C). This led us to monitor the levels of *Srg1-3* directly in the gut after systemic CDN stimulation. We found that depletion of dSTING in enterocytes led to dysregulation of *Srg1* as there was a modest increase in *Srg1* expression even in the absence of exogenous stimulation which was further enhanced by CDN stimulation (Fig 6D). This confirms cell-autonomous negative regulation of *Srg1* by dSTING in the gut epithelium. We also monitored intestinal *Srg2* and *Srg3* RNA expression. We found that CDN stimulation increased *Srg2* or *Srg3* RNA levels in the gut in a manner that is entirely dependent on dSTING expression in enterocytes (Fig 6E and 6F). This suggests that in response to CDNs the gut controls systemic expression of *Srg2*.

### Srg3 displays antiviral activity in response to systemic viral infection

**Fig 7:**
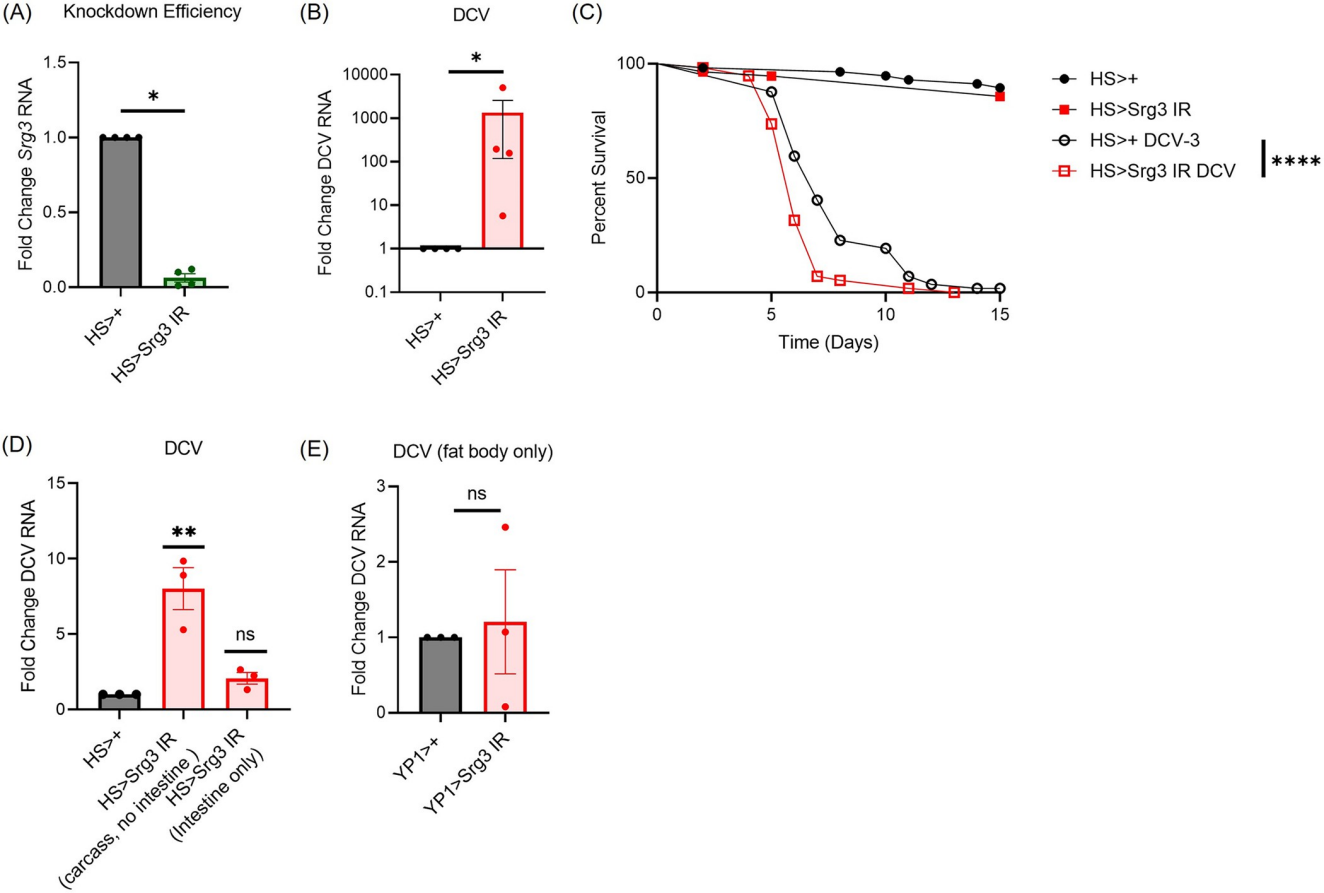
*Srg3* is antiviral against DCV infection. (A) Knockdown efficiency after heat shock mediated depletion of *Srg3* in whole animals. (B,D,E) Flies of the indicated genotype were infected with DCV for four days. Viral RNA from (B) 5 pooled whole animals or (D) 15 pooled fly carcasses without the intestine or 15 intestines and (E) 15 pooled fat bodies was quantified by RT-qPCR and normalized to controls relative to the housekeeping gene *rp49*. n = 3–4. Each dot represents an independent experiment with mean ± SEM shown. A one sample t-test was performed to determine statistical significance. (C) Survival of uninfected or DCV infected control (*HS>+*) and *Srg3* depleted (*HS>Srg3 IR*) animals. Four independent experiments were performed for a total of n = 153 uninfected and n = 154 infected flies across all four replicates. Significance was determined by a logrank test. ns, not significant, * p<0.05, ** p<0.01, **** p<0.0001.

Next, we explored the antiviral role of *Srg1*, *Srg2* and *Srg3* in DCV infection since systemic infection is controlled by CDNs and dSTING [14,17]. First, we confirmed efficient depletion of each gene using a ubiquitous heat shock driver (Figs 7A, S4A and S4C). Then we infected control (*HS>+*), *Srg1* depleted (*HS>Srg1 IR*) and *Srg2* depleted (*HS>Srg2 IR*) animals with DCV and quantified viral RNA levels at 4 days post infection by RT-qPCR. We found that loss of *Srg1* (*HS>Srg1 IR*) or *Srg2* (*HS>Srg2 IR*) had no impact on DCV infection as measured by RT-qPCR at day 4 post infection (S4B and S4D Fig). In contrast, when we challenged control (*HS>+*) or *Srg3* depleted (*HS>Srg3 IR*) flies with DCV, we found that loss of *Srg3* results in a significant increase in DCV infection in whole animals (Fig 7B). We also evaluated if *Srg3* influenced survival during DCV infection. Loss of *Srg3* does not impact the survival of uninfected flies but significantly decreased survival during DCV infection (Fig 7C).

As *Srg3* induction by systemic CDNs was partially controlled by dSTING in enterocytes, we challenged control (*HS>+*) and *Srg3* depleted (*HS>Srg3 IR*) flies with DCV and compared DCV RNA levels between the intestine and the remaining carcass without the gut. We found that systemic knockdown of *Srg3* led to a significant increase in DCV replication in the carcass but not the gut (Fig 7D). This suggests that non-intestinal sources of *Srg3* control DCV infection. Since we found that dSTING controlled *Srg3* induction in the fat body (Fig 5F), we tested if depletion of *Srg3* in the fat body impacted infection. We found that fat body specific loss of *Srg3* (*YP1>Srg3 IR*) did not impact DCV infection of the fat body (Fig 7E). Therefore, *Srg3* produced outside of the gut and fat body controls DCV infection. These data also suggest that dSTING controls DCV infection in the fat body through additional antiviral genes or mechanisms.

**Fig 8:**
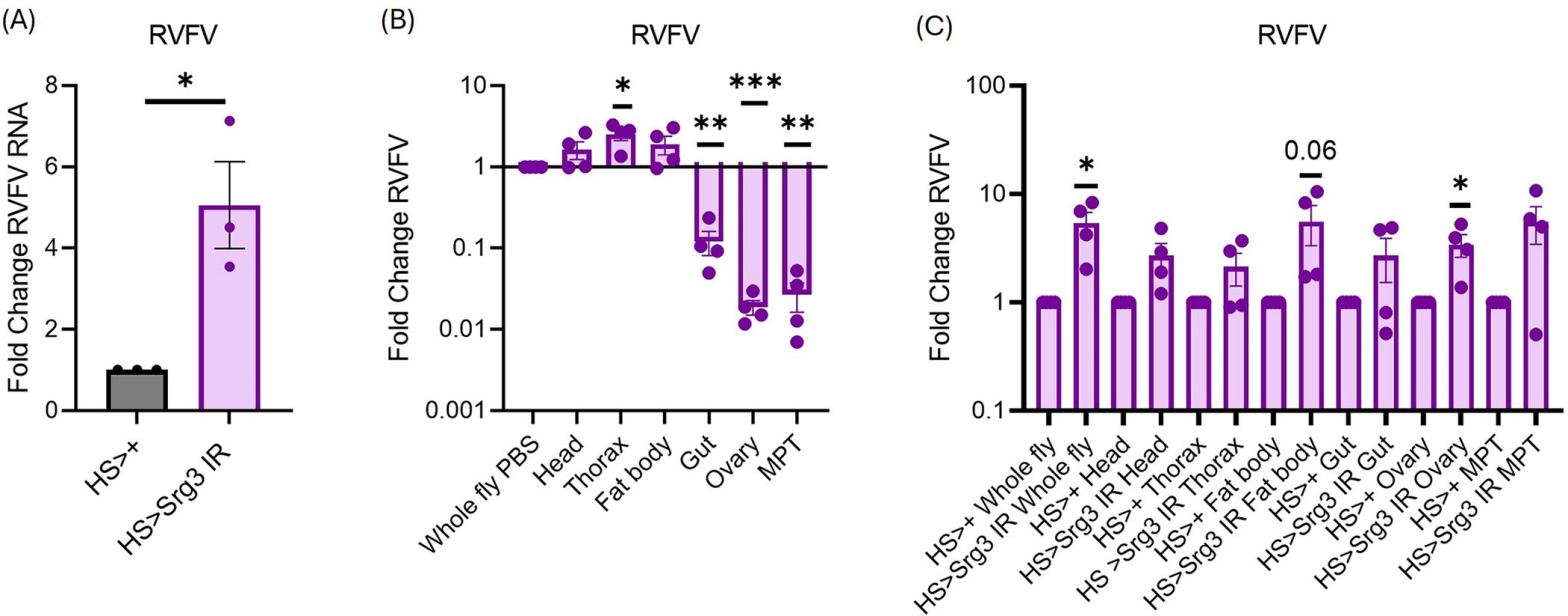
*Srg3* is antiviral against RVFV in ovarian tissue. (A-C) Control (*HS>+*) and *Srg3* depleted (*HS>Srg3 IR*) animals were infected with RVFV for seven days. Viral RNA was quantified from (A) 5 whole animals and (B,C) 5 whole animals or 15 pooled tissues (head, thorax, fat body, gut, ovary and Malpighian tubules (MPT)) by RT-qPCR. Viral RNA was normalized to (A,C) controls or (B) whole animals relative to the housekeeping gene *rp49*. n = 3–4. Each dot represents an independent experiment of 5 whole animals or 15 pooled tissues with mean ± SEM shown. A one sample t-test was performed to determine statistical significance. * p<0.05. ** p<0.01, ***p<0.001.

To determine the antiviral breadth of *Srg3*, we systemically challenged control (*HS>+*) and *Srg3* depleted (*HS>Srg3 IR*) flies with three distinct arthropod-borne viruses that infect humans: the alphavirus SINV, the bunyavirus RVFV, and the flavivirus DENV. We found that systemic depletion of *Srg3* led to a significant increase in RVFV infection (Fig 8A) but did not impact SINV or DENV infection (S5A and S5B Fig). This demonstrates specificity of the antiviral function of *Srg3*.

We further explored the antiviral activity of *Srg3* against RVFV. For these studies, we challenged control (*HS>+*) and *Srg3* (*HS>Srg3* IR) flies with RVFV and dissected individual tissues (head, thorax, fat body, gut, ovary, Malpighian tubules) to determine RVFV tropism and identify tissues in which *Srg3* is antiviral. We observed robust infection in the head, thorax and fat body and significantly less infection within the gut, ovary and Malpighian tubules (Fig 8B). We found that systemic depletion of *Srg3* led to a significant increase in viral infection in the ovaries and a trend toward increased infection in the fat body and other tissues (Fig 8C). These data suggest a potential role for *Srg3* in the ovaries for protection of the germline from vertically transmitted pathogens, including RVFV [26,27].

## Discussion

While ubiquitous loss of dSTING results in increased susceptibility to some systemic viral infections, less is known about how dSTING controls expression of antiviral gene programs for protection from infection in different tissues [14,16,17]. We found that loss of dSTING in the fat body led to increased systemic viral replication and decreased survival during DCV infection. We also found that loss of dSTING in visceral muscle and hemocytes led to increased viral replication during DCV infection. Our survival studies suggest that dSTING antiviral activity in the fat body is the most important for protection from viral lethality. The mechanism behind dSTING’s antiviral activity, specifically within the fat body, visceral muscle, and hemocytes is unclear as the canonical dSTING-controlled genes *Srg1* and *2* are not antiviral against DCV infection, and *Srg3* is not antiviral in the fat body. This suggests that a different dSTING induced gene, or that a combination of genes may mediate these antiviral effects.

We confirmed that dSTING is required for systemic CDN-induced gene expression changes and found that all tissues tested responded transcriptionally to CDN treatment [7,14,17]. However, when we explored the tissue-specific expression of the dSTING responsive *Srg1-3*, we found that the baseline expression of these genes varied across tissues. We also uncovered additional evidence of tissue specific control of the CDN-dSTING pathway. For example, we found that dSTING signaling in the fat body is the main source of CDN-induced *Srg1* RNA expression found in whole animals. In contrast, the source of CDN-induced *Srg2* RNA expression in whole animals is enterocytes and *Srg3* RNA is produced more ubiquitously. This is consistent with our previous data showing robust induction of *Srg2* and *Srg3* in the gut upon oral CDN treatment [17]. Altogether, this suggests that enterocytes are particularly poised to sense CDNs in the gut lumen and body cavity. *Srg2* is the ortholog of *SLC2A6*, a glucose transporter whose expression is also regulated by NF-kB in humans [33,34]. Since *Srg2* is likely a glucose transporter, this suggests that the antiviral CDN-dSTING pathway is connected to nutrient status in the intestine, which we previously found promotes local antiviral protection [35].

dSTING acts as a negative regulator of *Srg1* in the gut at homeostasis, but dSTING promotes *Srg1* expression at other body sites, suggesting the presence of additional gene regulators. Since NF-kB (Relish) is required for all CDN induced gene expression changes, the differences in *Srg1-3* expression across tissues suggests a role for tissue specific transcription factors [15]. Another possibility for this differential expression is the presence of negative regulators of CDNs, like the aldo-keto reductase enzyme RECON [36], in different tissues.

We found that DCV infection does not increase expression of the CDN-dSTING gene program at early timepoints which suggests that viral infection may not efficiently engage this pathway to drive differences in *Srg1-3* expression. Since it has been suggested that DCV recognition by cGLRs increases endogenous CDNs to activate dSTING and induce expression of *Srg1-3* [8], this data highlights the need for an *in vivo* tissue specific study of cGLR antiviral activity and production of CDNs at homeostasis and temporally throughout virus infection.

Additionally, gene programs that are induced by antiviral pathways are a rich source of antiviral effectors. Typically, many genes are induced, but only subsets are antiviral against any one virus. Consistent with this, we found that that *Srg1* and *Srg2* are dispensable during systemic DCV infection, but that *Srg3* decreased DCV replication and improved survival during DCV infection. *Srg3* is also selectively antiviral as it controls RVFV replication but has no effect on SINV or DENV infection. *Srg3* induction is controlled by dSTING in more than one body site, as loss of dSTING in enterocytes or the fat body results in loss of *Srg3* induction in each of these tissues, but not in the whole animal. *Srg3* expression in the fat body and enterocytes is dispensable in the control of DCV infection, however, loss of *Srg3* during RVFV infection led to increased infection of the ovaries. This is noteworthy as the ovary was less infected by RVFV compared to whole animals. This suggests that *Srg3* may be a major antiviral protector of the ovaries and may have an important role in the protection of the germline. This is particularly important to understand during RVFV infection as RVFV is transovarially transmitted in mosquito vectors as part of its natural cycle [26,27]; reviewed in [28]. An intervention that would block ovarian infection would have a major impact on RVFV transmission. *Srg3* is a 202 amino acid containing protein with an unknown molecular function which highlights the need for further studies to elucidate the mechanisms of *Srg3’s* antiviral activity.

In summary, we find that CDN-STING-gene induction and dSTING-mediated antiviral responses are different across tissues. Dissection of tissue-specific responses and the roles that STING and STING-induced genes play during viral infection is essential to enhance our understanding of how the innate immune system is orchestrated to control diverse viral infections. This understanding will inform design of tissue targeted therapeutics to boost specific immune responses to control pathogens. While we have begun to define the antiviral and tissue activity of genes induced by STING, future studies are needed to elucidate the mechanisms by which these genes regulate infection and immunity.

## Materials and methods

### Drosophila genetics

Flies were maintained on standard cornmeal food (Lab Express, #7005-NV) at room temperature. Female flies, 4–7 days old, of the indicated genotype were used for experiments. For all experiments, an independent experiment consisted of flies from independent crosses. Within an independent experiment 5–15 flies from the same progeny were experimentally manipulated and were then pooled together for analysis. Table 1 lists all fly stocks used in this study. Commercially available stocks were obtained from Bloomington Stock Center and Vienna Drosophila Resource Center. To heat shock animals, flies were placed in a 37°C incubator for 1 hour every day for three days prior to infection with virus, then heat shocked each day (skipping D2) post infection until collection.

**Table 1:** Genotypes used in present study.

Experimental Models: organisms and strains		
*D*. *melanogaster*: w1118	N. Perrimon (Harvard Medical School, Boston, MA	N/A
*D*. *melanogaster*: My01A-Gal4 (NP1-Gal4)	E. Baehrecke (University of Massachusetts Medical School, Worchester, MA)	FBtp0098092
*D*. *melanogaster*: HS-Gal4	Bloomington Drosophila Stock Center	FBst0001799; RRID:BDSC_1799
*D*. *melanogaster*: YP1-Gal4	Bloomington Drosophila Stock Center	Fban0002985
*D*. *melanogaster*: How24b-Gal4	Bloomington Drosophila Stock Center	FBrf0088875; RRID:BDSC_1767
*D*. *melanogaster*: Hml-Gal4	Bloomington Drosophila Stock Center	RRID:BDSC_6396
*D*. *melanogaster*: Act-Gal4	Bloomington Drosophila Stock Center	FBst0004414; RRID:BDSC_4414
*D*. *melanogaster*: UAS-STING IR (GD1905)	Vienna Drosophila Resource Center	FBst0463487; Cat#VDRC:4031
*D*. *melanogaster*: UAS-Relish IR (TRiP.HMS00070)	Bloomington Drosophila Stock Center	FBst0033661; RRID: BDSC_33661
*D*. *melanogaster*: UAS-Srg1 IR (GD6254)	Vienna Drosophila Resource Center	FBst0451566; Cat#VDRC:14717
*D*. *melanogaster*: UAS-Srg2 IR (GD2403)	Vienna Drosophila Resource Center	FBst0469670; Cat#VDRC:5203
*D*. *melanogaster*: UAS-Srg3 IR (KK112707)	Vienna Drosophila Resource Center	FBst0479362; Cat#VDRC:107542
*D*. *melanogaster*: UAS-Atg16 IR (TRiP.HMS01347)	Bloomington Drosophila Stock Center	FBst0034358; RRID:BDSC_34358
*D*. *melanogaster*: AGO2^414^	Kyoto Stock Center	FBst0313641; Cat#Kyoto:109027

### Virus propagation and infection

Viruses used in the present study included Drosophila C virus (DCV), Sindbis virus (SINVhrsp), dengue-2 virus S2 adapted (DENV-2) and Rift Valley fever virus (RVFV-MP12). DCV, SINV and DENV-2 were grown as previously described [37,38]. RVFV was grown in BHK cells as previously described [39].

For systemic treatments, an Eppendorf Femtojet was used to inoculate flies of the stated genotypes with 50nL vehicle (PBS) or 1 mg/mL 2’3’cGAMP (InvivoGen, #tlrl-nacga23s), 2’3’cdiAMP (InvivoGen, #vac-nacda2r) or virus as previously described [16,17,24,40]. Whole flies (n = 5), guts (n = 15), fat bodies (n = 15), heads (n = 15), ovaries (n = 15), Malpighian tubules (n = 15) and thorax (n = 15) were collected for each experiment. For virus challenge, flies were collected at 4dpi (DCV) or 7dpi (SINV, RVFV, DENV) to quantify viral RNA levels. Each experiment was performed at least 3 times as indicated.

For survival experiments, the number of dead flies were counted daily. At least 3 independent experiments per condition were performed with >15 flies per condition and data is aggregated.

### RNA extraction, reverse transcription, and Realtime qPCR

For total RNA extraction 5 whole flies, 15 guts, 15 thorax, 15 abdominal fat bodies, 15 heads, 15 ovaries, and 15 Malpighian tubules were pooled together and homogenized in TRIzol (Invitrogen), treated with DNase 1, and purified using an RNA Clean and Concentrator Kit (Zymo Research) according to the manufacturers’ protocol. cDNA was generated using the SuperScript III cDNA synthesis kit (Invitrogen). In three independent experiments cDNA was analyzed in triplicate with gene specific primers and SYBR Green PCR Master Mix (Applied Biosystems). Data was normalized to *rp49* by relative quantification using the ddCT method. Primers are listed in Table 2.

**Table 2:** Quantitative Real-Time PCR primers.

Primer Name	Sequence (5’-> 3’)	Source
RP49 Forward	AAGAAGCGCACCAAGCACTTCATC	[41]
RP49 Reverse	TCTGTTGTCGATACCCTTGGGCTT	[41]
Sindbis Forward	GCTGAAACACCATCGCTCTGCTTT	[41]
Sindbis Reverse	TGGTGTCGAAGCCAATCCACTACA	[41]
DCV Forward	TGGGACAGGCAGTTAATTCGTCCA	[41]
DCV Reverse	AAGACCGCAGTGTCTACACCACAT	[41]
DENV Forward	TGAGGACTACATGGGCTCTG	[42]
DENV Reverse	AAACCTCCCTGGATTTCCTT	[42]
RVFV Forward	CAAGCAGTGGACCGCAATGAGA	[42]
RVFV Reverse	GGGCTTGTTGCCACGAGTTAGA	[42]
Srg1 Forward	GTGTCCATTATCCGCACAAG	[14]
Srg1 Reverse	ACTGGGGTATCTGACGGATG	[14]
Srg2 Forward	GCGTTTTGGCCCTTATTATG	[14]
Srg2 Reverse	CTTTTGTAGCCGACGCAGTG	[14]
Srg3 Forward	GCGACCGTCATTGGATTGG	[14]
Srg3 Reverse	TGATGGTCCCGTTGATAGCC	[14]
dSTING Forward	GGCTGTGGATAATCCTGCGG	[16]
dSTING Reverse	ACGTTCCTGTAATTCCCGGTAA	[16]

### Quantification and statistical analysis

P values for RT-qPCR experiments were obtained by performing a two-tailed t-test or one-way Anova with multiple comparisons and correction for multiple tests on ddCT values from at least three independent experiments. A Grubbs’ test was performed to remove statistical outliers. For survival experiments results are presented as a Kaplan-Meier survival curve and a log-rank test was performed to determine significance. Visualization of data was performed in Prism 8 (Graphpad). The statistical parameters for experiments can be found in the figure legends, n indicates independent experiments. The number of animals used per experiment can be found in the Virus Propagation and Infection section.

## Supporting information

S1 Fig CDN induced gene expression changes are ATG16- and Ago2-independent. (A) Survival curves for uninfected or DCV infected control (*How24b>+*) and *dSTING* visceral muscle depleted (*How24b>dSTING IR*) flies. Three independent experiments were performed for a total of n = 79 uninfected and n = 80 infected flies across all three replicates. Significance was determined by a logrank test. ns, not significant. (B) Control (*Act>+*) and *Atg16* knockdown (*Act>Atg16 IR*) flies and (C) control (w1118) or *Ago2* mutant *(Ago2* -/-) flies were treated with 2’3’cGAMP. *Srg2* expression was quantified by RT-qPCR and normalized to controls relative to housekeeping gene *rp49*. n = 3. Each dot represents an independent experiment of 5 pooled whole flies with mean ± SEM shown. A one-way Anova with multiple comparisons was performed to determine statistical significance. ns, not significant, * p<0.05.
(TIF)

S2 Fig 2’3cdiAMP induces *Srg1-3* across tissues. (A-D) Wild type (w1118) flies were systemically treated with either PBS or 2’3’cGAMP and 5 pooled whole flies or groups of 15 flies were dissected for specific tissues (head, thorax, fat body, gut, ovary, Malpighian tubules (MPT)). 6 hours later, indicated genes (*Srg1-3*, *dSTING*) were quantified by RT-qPCR and normalized to PBS treated tissues relative to *rp49*. n = 2–3. Each dot represents an independent experiment with mean ± SEM shown. A one sample t-test was performed to determine statistical significance. * p<0.05, ** p<0.01.
(TIF)

S3 Fig Systemic DCV infection does not induce *Srg1-3* or *dSTING* at 6hr or 24hr post infection. (A-H) Wild type (w1118) flies were systemically challenged with either PBS or DCV and 5 pooled whole flies or groups of 15 flies were dissected for specific tissues (head, thorax, fat body, gut, ovary, Malpighian tubules (MPT)) 6 or 24 hours later. Indicated genes (*Srg1-3*, *dSTING*) were quantified by RT-qPCR and normalized to PBS injected flies relative to *rp49*. n = 3. Each dot represents an independent experiment of 5 pooled flies or 15 pooled tissues with mean ± SEM shown. A one sample t-test was performed to determine statistical significance. * p<0.05. (C-H) no comparisons significant.
(TIF)

S4 Fig *Srg1* and *Srg2* are not antiviral against DCV infection. (A,C) Knockdown efficiency after heat shock mediated depletion of (A) *Srg1* or (C) *Srg2*. (B,D) Control *(HS>+*) and (B) *Srg1* depleted (*HS>Srg1 IR*) or (D) *Srg2* depleted (*HS>Srg2 IR*) flies were infected with DCV. Virus and indicated gene levels were quantified by RT-qPCR and normalized to controls relative to the housekeeping gene *rp49*. n = 3. Each dot represents an independent experiment of 5 pooled whole flies with mean ± SEM shown. A one sample t-test was performed to determine statistical significance. ns, not significant, * p<0.05, ** p<0.01.
(TIF)

S5 Fig *Srg3* is not antiviral against SINV or DENV infection. (A,B) Control *(HS>+*) and *Srg3* depleted flies (*HS>Srg3 IR*) were systemically infected with (A) SINV or (B) DENV for 7 days. Virus RNA levels were quantified by RT-qPCR and normalized to controls relative to the housekeeping gene *rp49*. n = 3. Each dot represents an independent experiment of 5 pooled whole flies with mean ± SEM shown. A one sample t-test was performed to determine statistical significance. ns, not significant.
(TIF)
