# Supplementary figures and images for: Tissue specific innate immune responses impact viral infection in Drosophila
Source: PLoS Pathog. 2024 Nov 4;20(11):e1012672. doi: 10.1371/journal.ppat.1012672 (PMC11563389; doi:10.1371/journal.ppat.1012672)

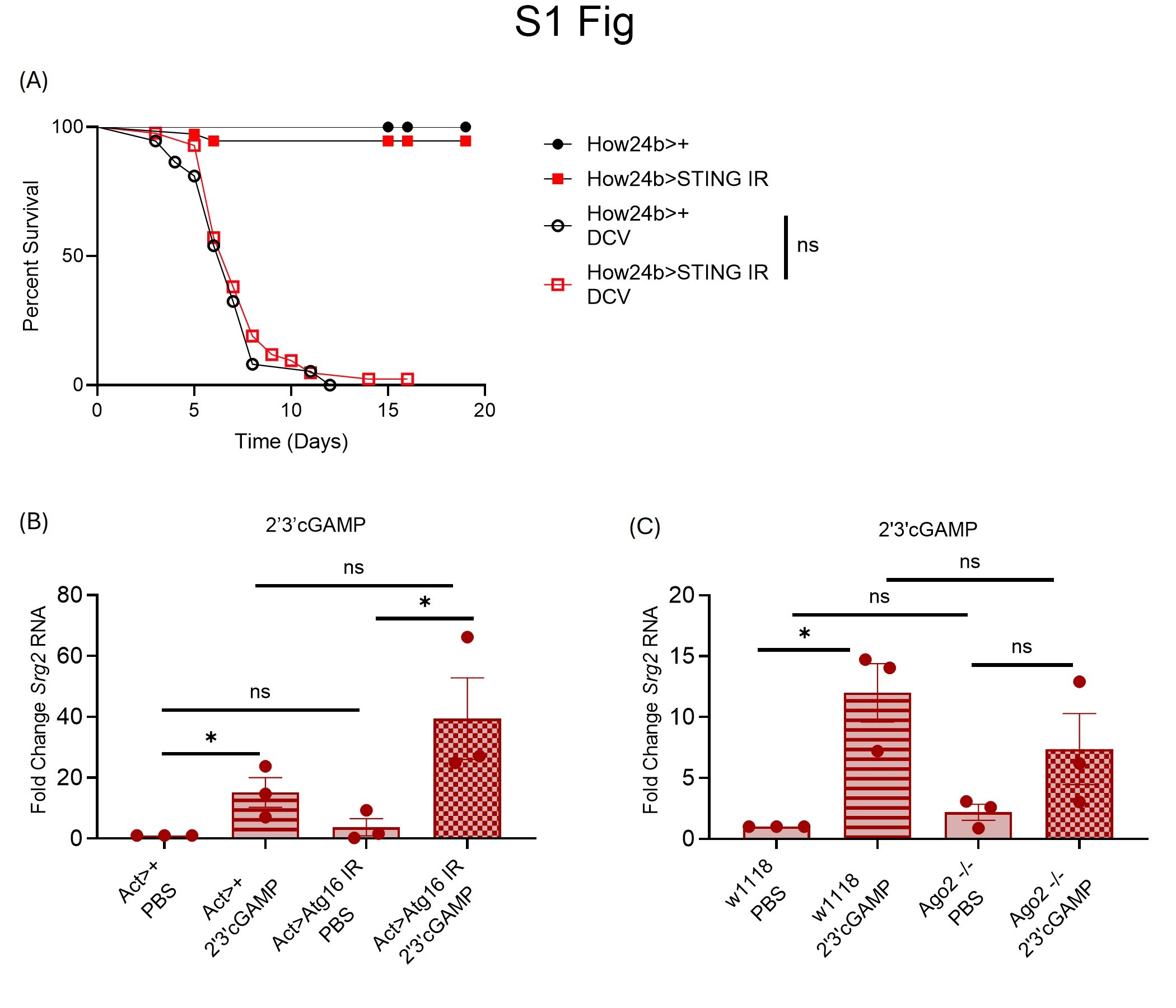

Supplement: S1 Fig — (A) Survival curves for uninfected or DCV infected control (How24b>+) and dSTING visceral muscle depleted (How24b>dSTING IR) flies. Three independent experiments were performed for a total of n = 79 uninfected and n = 80 infected flies across all three replicates. Significance was determined by a logrank test. ns, not significant. (B) Control (Act>+) and Atg16 knockdown (Act>Atg16 IR) flies and (C) control (w1118) or Ago2 mutant (Ago2 -/-) flies were treated with 2’3’cGAMP. Srg2 expression was quantified by RT-qPCR and normalized to controls relative to housekeeping gene rp49. n = 3. Each dot represents an independent experiment of 5 pooled whole flies with mean ± SEM shown. A one-way Anova with multiple comparisons was performed to determine statistical significance. ns, not significant, * p<0.05. (TIF) [file ppat.1012672.s001.tif]

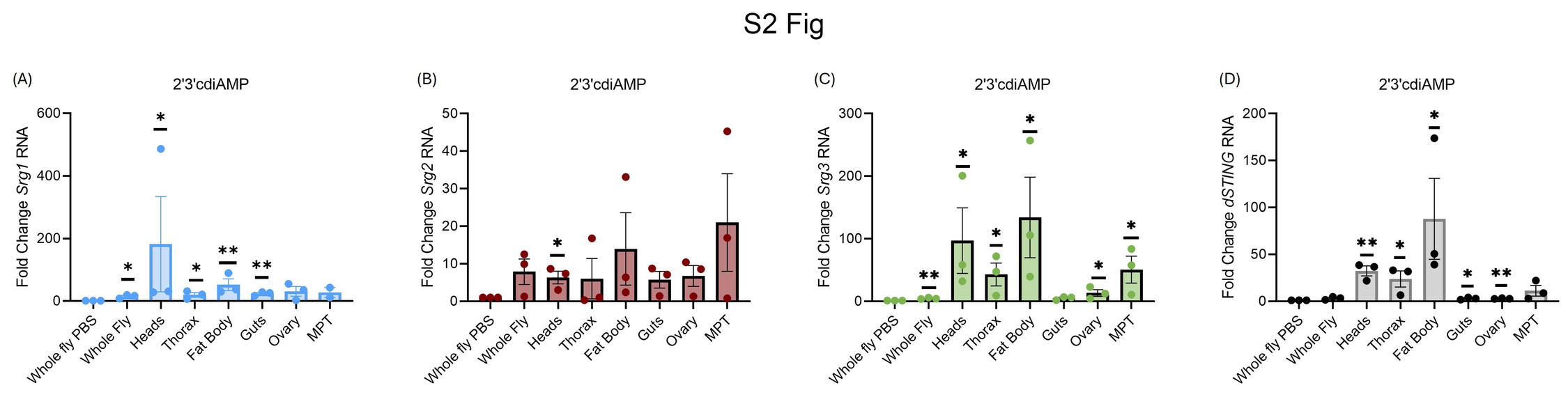

Supplement: S2 Fig — (A-D) Wild type (w1118) flies were systemically treated with either PBS or 2’3’cGAMP and 5 pooled whole flies or groups of 15 flies were dissected for specific tissues (head, thorax, fat body, gut, ovary, Malpighian tubules (MPT)). 6 hours later, indicated genes (Srg1-3, dSTING) were quantified by RT-qPCR and normalized to PBS treated tissues relative to rp49. n = 2–3. Each dot represents an independent experiment with mean ± SEM shown. A one sample t-test was performed to determine statistical significance. * p<0.05, ** p<0.01. (TIF) [file ppat.1012672.s002.tif]

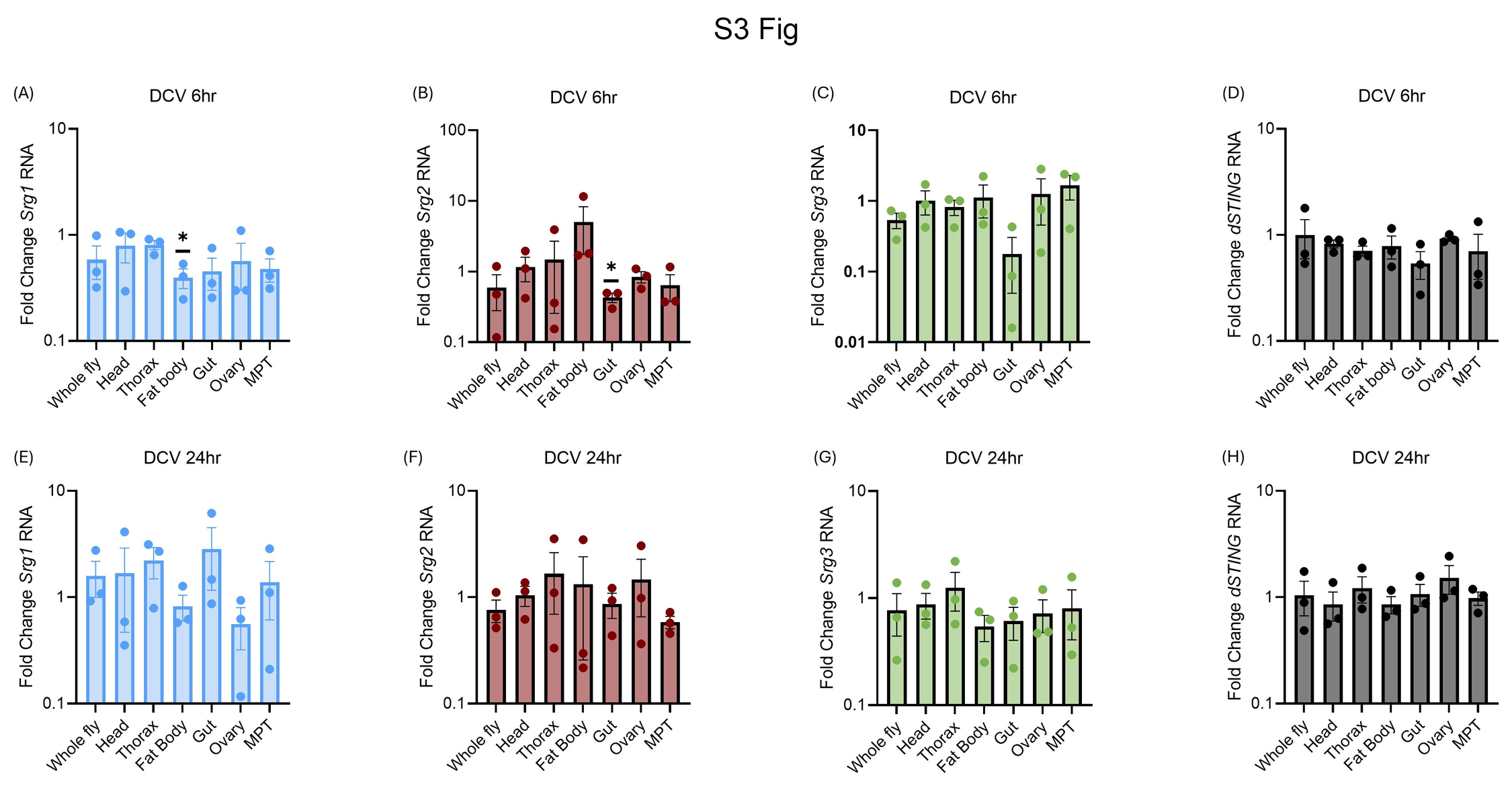

Supplement: S3 Fig — (A-H) Wild type (w1118) flies were systemically challenged with either PBS or DCV and 5 pooled whole flies or groups of 15 flies were dissected for specific tissues (head, thorax, fat body, gut, ovary, Malpighian tubules (MPT)) 6 or 24 hours later. Indicated genes (Srg1-3, dSTING) were quantified by RT-qPCR and normalized to PBS injected flies relative to rp49. n = 3. Each dot represents an independent experiment of 5 pooled flies or 15 pooled tissues with mean ± SEM shown. A one sample t-test was performed to determine statistical significance. * p<0.05. (C-H) no comparisons significant. (TIF) [file ppat.1012672.s003.tif]

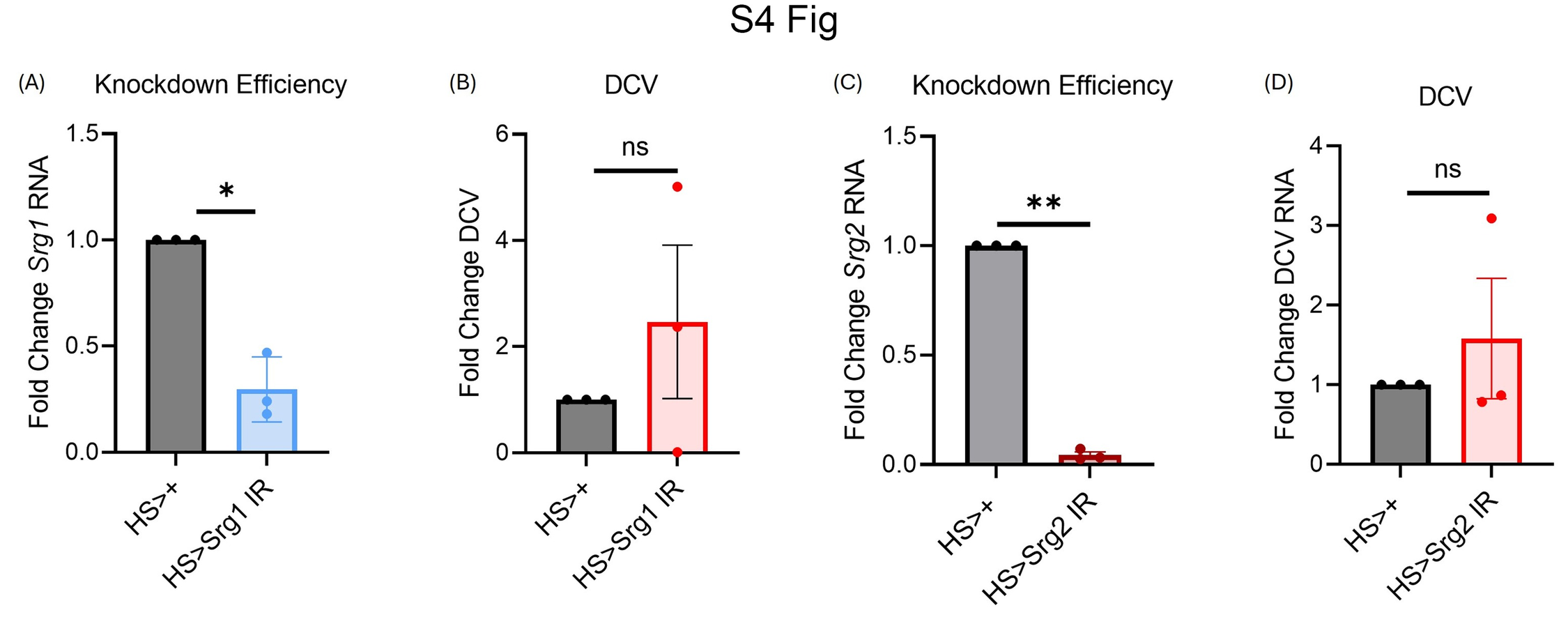

Supplement: S4 Fig — (A,C) Knockdown efficiency after heat shock mediated depletion of (A) Srg1 or (C) Srg2. (B,D) Control (HS>+) and (B) Srg1 depleted (HS>Srg1 IR) or (D) Srg2 depleted (HS>Srg2 IR) flies were infected with DCV. Virus and indicated gene levels were quantified by RT-qPCR and normalized to controls relative to the housekeeping gene rp49. n = 3. Each dot represents an independent experiment of 5 pooled whole flies with mean ± SEM shown. A one sample t-test was performed to determine statistical significance. ns, not significant, * p<0.05, ** p<0.01. (TIF) [file ppat.1012672.s004.tif]

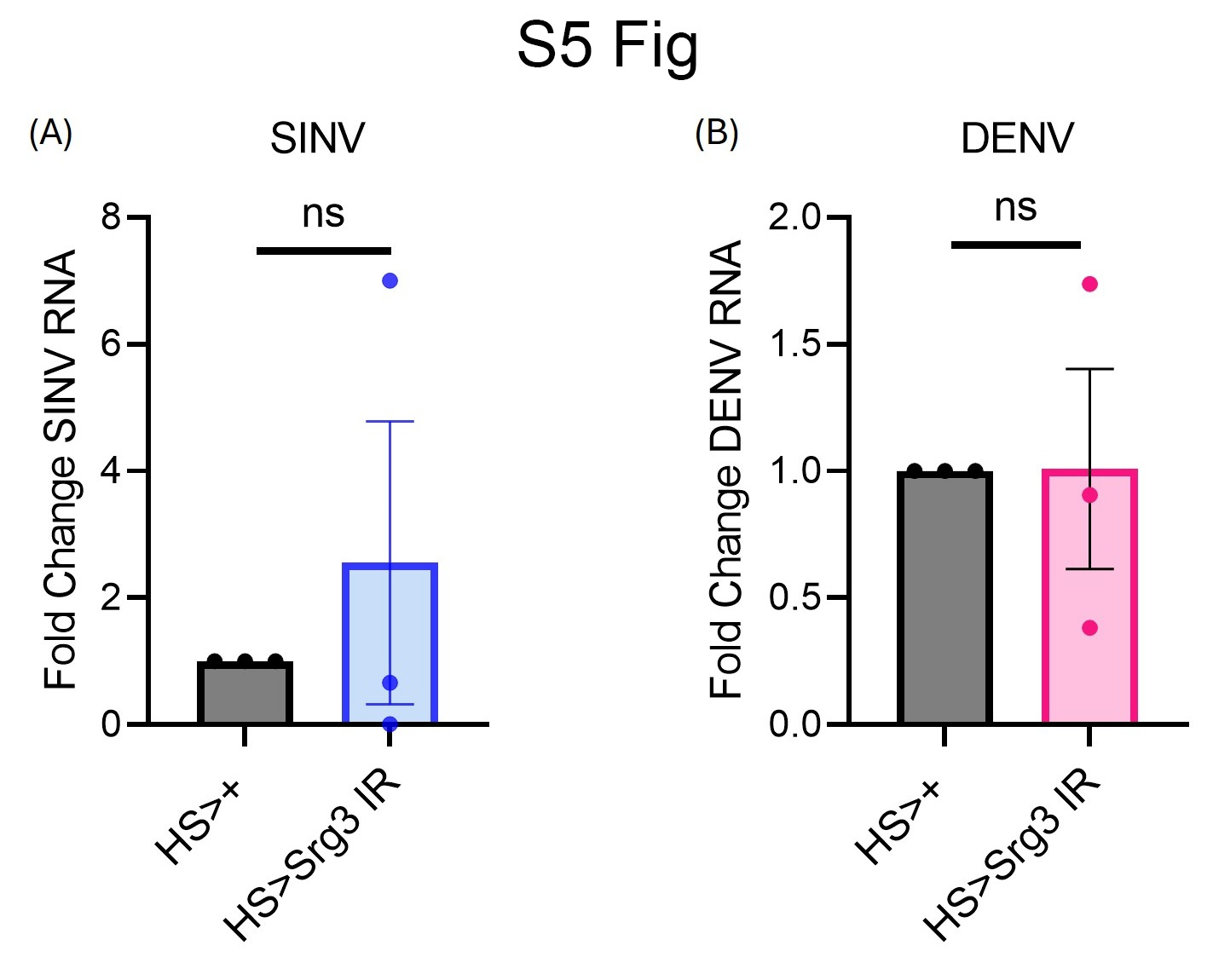

Supplement: S5 Fig — (A,B) Control (HS>+) and Srg3 depleted flies (HS>Srg3 IR) were systemically infected with (A) SINV or (B) DENV for 7 days. Virus RNA levels were quantified by RT-qPCR and normalized to controls relative to the housekeeping gene rp49. n = 3. Each dot represents an independent experiment of 5 pooled whole flies with mean ± SEM shown. A one sample t-test was performed to determine statistical significance. ns, not significant. (TIF) [file ppat.1012672.s005.tif]
